# Sustained effect of Scotland's HIV pre‐exposure prophylaxis programme on transmission among gay and bisexual men who have sex with men: Population‐based retrospective cohort study

**DOI:** 10.1111/hiv.70255

**Published:** 2026-05-08

**Authors:** Alan Yeung, Claudia Estcourt, Beth Cullen, Rebecca Metcalfe, Alison Rodger, Dan Clutterbuck, Nicola Steedman, Rak Nandwani, Duncan McCormick, Sharon Hutchinson

**Affiliations:** ^1^ School of Health and Life Sciences Glasgow Caledonian University Glasgow UK; ^2^ Public Health Scotland Glasgow UK; ^3^ NHS Greater Glasgow and Clyde Glasgow UK; ^4^ Institute for Global Health University College London London UK; ^5^ NHS Lothian Edinburgh UK; ^6^ Scottish Government Edinburgh UK

**Keywords:** epidemiology, GBMSM, HIV pre‐exposure prophylaxis, incidence, prevention

## Abstract

**Objectives:**

To evaluate the population‐level impact of seven years of Scotland's HIV pre‐exposure prophylaxis (PrEP) programme among gay, bisexual and other men who have sex with men (GBMSM).

**Methods:**

National administrative data were used to estimate PrEP coverage and HIV incidence among 35 342 GBMSM attending specialist sexual health services between July 2015 and March 2024. Poisson regression was used to investigate the risk of HIV acquisition and its association with the extent of PrEP prescribing.

**Results:**

PrEP coverage increased from 21% in the initial PrEP period (July 2017 to March 2020) to 45% in the latter period (April 2022 to March 2024). HIV incidence reduced from 4.46 per 1000 person‐years in the pre‐PrEP period (July 2015 to June 2017) to 1.66 in the initial PrEP period (aIRR 0.37, 95% CI 0.25–0.54) and remained stable thereafter. During the entire PrEP period, the risk of HIV acquisition was reduced among those ‘currently prescribed’ (involving 4 HIV acquisitions among 11 720 person‐years) compared to those never prescribed (60 HIV acquisitions among 34 328 person‐years) (aIRR 0.19, 95% CI 0.07–0.51). Compared to those currently prescribed PrEP, risk was greatest in those prescribed 12+ months ago (11 HIV acquisitions among 3824 person‐years) (aIRR 8.13, 95% CI 2.58–25.64).

**Conclusions:**

HIV incidence was sustained at a low level as PrEP coverage broadened among GBMSM, but did not reduce further. Efforts to support persistence and re‐engagement with PrEP are warranted in those at ongoing risk of HIV.

## INTRODUCTION

Pre‐exposure prophylaxis (PrEP) is a cornerstone of HIV prevention worldwide, whereby individuals who are HIV negative but at increased risk of acquisition take antiretroviral medication to prevent infection [1, 2]. The number of people using oral PrEP worldwide has risen from approximately 200 000 in 2017 to 3.5 million in 2023, but falls far short of the global 2025 target of 21.2 million [3]. Oral PrEP is more than 99% effective at preventing HIV acquisition and can be taken daily or around the time of sex (event‐based) [4]. Among gay, bisexual and other men who have sex with men (GBMSM), oral PrEP has been associated with an 87% reduction in HIV incidence under open‐label trial conditions in England [5]. In real‐world conditions, effectiveness has been estimated at 75% in Scotland [6] and 60% in one study from France [7].

Scotland was one of the first countries to adopt a national PrEP programme, in July 2017, enabling free oral HIV PrEP (involving antiretroviral medications tenofovir disoproxil and emtricitabine) prescribed from specialist sexual health clinics to adults meeting risk‐based eligibility criteria [8]. While eligibility has since been extended beyond the initial criteria [4], over 90% of PrEP is prescribed for GBMSM. This reflects a higher coverage for this group compared to other at‐risk populations, in the context that fewer new HIV diagnoses involving recently acquired infection (54% between 2018 and 2024) were attributed to GBMSM [9].

Few studies have investigated the real‐world uptake of PrEP and its impact on transmission among GBMSM [5, 10, 11, 12]. Furthermore, it is unclear whether the effect of PrEP in reducing HIV incidence at the population level has been maintained as the intervention is broadened to a wider group or affected by the COVID‐19 pandemic [13]. Additionally, high rates of PrEP discontinuation and sub‐optimal adherence have been reported across regions, but the impact of this on HIV transmission is unclear [14, 15, 16, 17].

Using national data collected from specialist sexual health services in Scotland, we aimed to (i) examine PrEP coverage among GBMSM over the first 7 years of the Scottish programme (according to calendar periods prior to, during and after the COVID‐19 pandemic), and (ii) investigate the impact of PrEP on the incidence of HIV acquisition among GBMSM over these periods. As each calendar period has varying levels of PrEP coverage, this analysis allows the impact of different uptake on HIV incidence to be assessed. To gauge the impact of discontinuation, we further examined the incidence of HIV acquisition according to periods of elapsed time since the last PrEP prescription.

## MATERIALS AND METHODS

### Data sources and cohort

The National Sexual Health System (NaSH) is a clinical electronic records system used by specialist sexual health services in mainland Scotland (accounting for geographical areas covering >98% of the population) since 2011 [18]. A retrospective cohort of GBMSM aged over 15 years was formed by extracting records for individuals assigned male sex at birth who reported at least one male sexual partner and had attended a sexual health clinic during the study period from July 2015 to March 2024 (with the period from July 2015 to June 2017 representing a pre‐PrEP period). Individuals with an earlier HIV diagnosis (i.e. positive test prior to their first attendance in the study period) were excluded.

NaSH was used to obtain data on patient characteristics, attendances, PrEP prescriptions (specified as duration in months for those prescribed daily use and number of pills for event‐based use), HIV post‐exposure prophylaxis (PEP) prescriptions (used to prevent HIV acquisition *after* a potential exposure) and laboratory test results for HIV and bacterial STIs (gonorrhoea and chlamydia at any anatomical site); data on infectious syphilis diagnoses were not available on NaSH.

Characteristics available included age, sex, NHS board of residence, ethnicity (‘White British’ including White with unspecified nationality, ‘White Other’ and ‘Ethnic Minority Groups’) and socioeconomic status (measured by the Scottish Index of Multiple Deprivation), history of injecting drugs and recent STI diagnoses/HIV PEP use (for the period from 12 months prior to 3 months post‐first clinic attendance within each calendar period). The latter data were used in combination as an indicator of sexual risk for HIV acquisition, assigned hierarchically in the order of (i) those prescribed HIV PEP and/or with a rectal bacterial STI diagnosis, (ii) those with a non‐rectal bacterial STI diagnosis and (iii) those not diagnosed with a bacterial STI or prescribed HIV PEP.

### PrEP coverage

PrEP coverage was defined as the number of GBMSM prescribed PrEP at least once as a proportion of GBMSM who attended a sexual health clinic during three sub‐periods: ‘PrEP‐period‐1’ (July 2017–March 2020), ‘PrEP‐period‐2’ (April 2020–March 2022, relating to the COVID‐19 pandemic) and ‘PrEP‐period‐3’ (April 2022–March 2024). Logistic regression was used to assess factors associated with being prescribed PrEP, stratified by these sub‐periods; adjusted odds ratios (aORs) were obtained accounting for the aforementioned characteristics.

### HIV incidence

A sub‐cohort involving those who initially tested HIV negative and with at least one further test on NaSH during the study period was used to estimate HIV incidence, as described elsewhere [6]. Person‐years (PY) were calculated from the date of the first negative HIV test (or from the start of the study period for those who had tested negative in the prior two years) up to either their last negative test or their first positive HIV test (regarded as an ‘incident infection’) in the study period. Incidence of HIV was calculated as the rate per 1000 PY, with 95% confidence intervals (CIs) estimated assuming a Poisson distribution.

### Impact of PrEP

Two analyses were performed to investigate the impact of PrEP on HIV incidence. First, examining incidence rates temporally according to four sub‐periods—the three sub‐periods mentioned previously and a ‘pre‐PrEP‐period’ (July 2015–June 2017). Incidence rate ratios (IRRs) and adjusted incidence rate ratios (aIRRs) were estimated using Poisson regression.

The second analysis also used Poisson regression but the analysis was confined to the PrEP period (July 2017–March 2024) and compared HIV incidence according to PrEP status, defined as ‘currently prescribed’, ‘previously prescribed’ or ‘never prescribed’. Currently prescribed assumed that PrEP would last for the exact duration prescribed for daily use (77.6% of all prescriptions, Table S1); where event‐based dosing was indicated, it was assumed that patients used four pills each week (in‐line with one event‐based dose per week) [19].

We hypothesized that those with a longer duration since their last PrEP prescription would be more likely to have stopped using PrEP. Therefore, we also examined the risk of HIV acquisition according to increasing time elapsed since the last PrEP prescription to assess the ongoing risk for people who may have discontinued PrEP. Sensitivity analyses were conducted which assumed different patterns of PrEP use: (i) 5 pills per week for daily and 2 pills per week for event‐based prescriptions [20], (ii) daily use for all PrEP prescriptions, and (iii) four PrEP pills each week for all prescriptions.

### Ethics statement

We conducted secondary analysis of pseudonymized administrative healthcare data for which pan‐Scotland Caldicott approval is in place [21]. Formal ethical approval was not required.

## RESULTS

Over the study period, 35 342 GBMSM attended sexual health clinics, ranging from 13 257 to 20 674 across the pre‐PrEP and PrEP periods 1–3 (Table 1). The proportion of new attendees was similar in the pre‐PrEP period and PrEP period 1 (around 40%) but reduced to around 25% in the PrEP periods 2 and 3. The median age increased from 29 (IQR 23–40) in the pre‐PrEP period to 32 (IQR 26–43) years in PrEP period 3. The proportions by other characteristics remained relatively stable over time: people of minority ethnic groups (6%–7%), from the two largest NHS board regions (Greater Glasgow and Clyde or Lothian, approximately 60%), from areas of the two most deprived quintiles (quintiles 1–2, 41%–43%), and reported ever injecting drugs (2%). The proportion prescribed HIV PEP and/or with a rectal STI diagnosis in the previous year increased from 13% pre‐PrEP to 18% in PrEP period 3. Most of this increase was attributable to increased numbers diagnosed with a rectal STI (Table S2).

**TABLE 1:** Characteristics of 35 342 GBMSM who attended sexual health clinics (including remote attendances^e^) in Scotland between July 2015 and March 2024 by analysis period.

	Pre‐PrEP period (July 2015 to June 2017) *n* (col %)	PrEP period 1 (July 2017 to March 2020) *n* (col %)	PrEP period 2 (April 2020 to March 2022) *n* (col %)	PrEP period 3 period (April 2022 to March 2024) *n* (col %)
Total^a^	14 752	20 674	13 257	16 832
Attendance history^b^				
Last attended within the previous 2 years	5587 (37.9%)	7126 (34.5%)	6626 (50.0%)	7935 (47.1%)
Last attended more than 2 years ago	2997 (20.3%)	4647 (22.5%)	3214 (24.2%)	4808 (28.6%)
Never attended previously	6168 (41.8%)	8901 (43.1%)	3417 (25.8%)	4089 (24.3%)
Age				
Median (IQR)	29 (23–40)	29 (24–40)	31 (25–41)	32 (26–43)
<24	4749 (32.2%)	6186 (29.9%)	3139 (23.7%)	3355 (19.9%)
25–39	6247 (42.3%)	9293 (45.0%)	6504 (49.1%)	8422 (50.0%)
40–49	1969 (13.3%)	2527 (12.2%)	1752 (13.2%)	2462 (14.6%)
50+	1787 (12.1%)	2668 (12.9%)	1862 (14.0%)	2593 (15.4%)
Ethnicity^c^				
White British	10 574 (71.7%)	13 617 (65.9%)	8449 (63.7%)	10 485 (62.3%)
White other	1313 (8.9%)	1651 (8.0%)	889 (6.7%)	1052 (6.2%)
Minority ethnic groups	936 (6.3%)	1331 (6.4%)	942 (7.1%)	1211 (7.2%)
Not known	1929 (13.1%)	4075 (19.7%)	2977 (22.5%)	4084 (24.3%)
NHS board of residence				
Greater Glasgow and Clyde	4841 (32.8%)	6585 (31.9%)	4372 (33.0%)	5355 (31.8%)
Lothian	4074 (27.6%)	5613 (27.2%)	3582 (27.0%)	4347 (25.8%)
Rest of Scotland	5837 (39.6%)	8476 (41.0%)	5303 (40.0%)	7130 (42.4%)
Deprivation quintile of residence				
1–2 (most deprived)	6121 (41.5%)	8476 (41.0%)	5713 (43.1%)	7088 (42.1%)
3–5	7931 (53.8%)	11 064 (53.5%)	7135 (53.8%)	9152 (54.4%)
Not known	700 (4.7%)	1134 (5.5%)	409 (3.1%)	592 (3.5%)
Ever injected drugs				
No	13 461 (91.2%)	18 462 (89.3%)	11 881 (89.6%)	15 054 (89.4%)
Yes	332 (2.3%)	408 (2.0%)	289 (2.2%)	306 (1.8%)
Not known	959 (6.5%)	1804 (8.7%)	1087 (8.2%)	1472 (8.7%)
Recent STI/PEP history^d^				
Diagnosed with a rectal STI and/or prescribed HIV PEP	1905 (12.9%)	2855 (13.8%)	2093 (15.8%)	3074 (18.3%)
Diagnosed with an STI (non‐rectal)	1096 (7.4%)	1449 (7.0%)	803 (6.1%)	1375 (8.2%)
Not diagnosed with an STI or prescribed PEP	11 751 (79.7%)	16 370 (79.2%)	10 361 (78.2%)	12 383 (73.6%)

^a^ This excludes those who tested positive for HIV prior to their first attendance in the pre‐PrEP period (*n* = 279), the pre‐pandemic period (*n* = 405), the pandemic period (*n* = 429) and the post‐pandemic period (*n* = 533).

^b^ Relates to the period prior to a patient's first attendance within each analysis period dating back to the commencement of NaSH in 2008.

^c^ White British includes White with unspecified nationality; White Other includes minority White ethnic groups and people of non‐British White ethnicity.

^d^ Within a year prior to the date of first attendance in a period or up to 3 months beyond the date of first attendance. STI only includes gonorrhoea and chlamydia. Patients have been assigned to only one category based on their highest risk factor.

^e^ HIV and sexual health services were extensively redesigned during and after the COVID‐19 pandemic, with some elements of service provision now being delivered remotely online. In addition, innovative means of delivering PrEP are being implemented with a view to achieving HIV transmission elimination. These developments have impacted the way in which people access HIV PrEP in Scotland.

### PrEP coverage and associated factors

Over the duration of the PrEP programme (July 2017–March 2024), 10 699 of 30 712 GBMSM that attended sexual health clinics (35%) were prescribed PrEP at least once. PrEP coverage increased from 21% in PrEP period 1 to 36% in PrEP period 2 and 45% in PrEP period 3 (among patients attending within each calendar period) and this was reasonably consistent across different characteristics (Figure S1). Notably, PrEP coverage increased particularly between PrEP periods 1 and 3 among those prescribed HIV PEP and/or with a rectal STI diagnosis (from 40% to 60%, respectively) and people of minority ethnic groups (from 28% to 60%, respectively).

Several factors showed consistent associations with being prescribed PrEP over time (Table 2). Those who last attended over 2 years ago or had never previously attended were less likely than patients who had attended within the previous 2 years to be prescribed PrEP (aOR 0.36, 95% CI 0.33–0.39 and aOR 0.80, 95% CI 0.74–0.88, respectively, in PrEP period 3). Patients of minority ethnic groups were more likely to be prescribed PrEP compared to patients of White British ethnicity (aOR 1.25, 95% CI 1.09–1.42 in PrEP period 3), while patients of White Other ethnicity were less likely to be prescribed PrEP (aOR 0.78, 95% CI 0.68–0.89 in PrEP period 3).

**TABLE 2:** Extent of, and factors associated with, PrEP uptake among GBMSM attending sexual health clinics in Scotland, stratified by analysis period.

	PrEP period 1 July 2017 to March 2020		PrEP period 2 April 2020 to March 2022		PrEP period 3 April 2022 to March 2024	
	Prescribed PrEP (% Uptake)^d^	aOR (95% CI)^e^	Prescribed PrEP (% Uptake)^d^	aOR (95% CI)^e^	Prescribed PrEP (% Uptake)^d^	aOR (95% CI)^e^
Total	4336 (21.0%)		4728 (35.7%)		7650 (45.4%)	
Attendance history^a^						
Last attended within the previous 2 years	2133 (29.9%)	1.00	3054 (46.1%)	1.00	4489 (56.6%)	1.00
Last attended more than 2 years ago	896 (19.3%)	0.57 (0.52–0.63)	757 (23.6%)	0.36 (0.32–0.39)	1497 (31.1%)	0.36 (0.33–0.39)
Never attended previously	1307 (14.7%)	0.57 (0.52–0.62)	917 (26.8%)	0.66 (0.60–0.73)	1664 (40.7%)	0.80 (0.74–0.88)
Age						
Median (IQR)	30 (25–40)		32 (26–41)		32 (26–42)	
<24	1051 (17.0%)	1.00	940 (29.9%)	1.00	1407 (41.9%)	1.00
25–39	2153 (23.2%)	1.42 (1.30–1.55)	2451 (37.7%)	1.33 (1.21–1.47)	4008 (47.6%)	1.27 (1.16–1.39)
40–49	633 (25.0%)	1.65 (1.46–1.86)	722 (41.2%)	1.47 (1.29–1.68)	1186 (48.2%)	1.29 (1.15–1.44)
50+	499 (18.7%)	1.19 (1.05–1.34)	615 (33.0%)	1.04 (0.91–1.19)	1049 (40.5%)	0.93 (0.83–1.05)
Ethnicity^b^						
White British	3226 (23.7%)	1.00	3367 (39.9%)	1.00	5135 (49.0%)	1.00
White Other	319 (19.3%)	0.78 (0.68–0.89)	325 (36.6%)	0.80 (0.69–0.93)	505 (48.0%)	0.82 (0.72–0.93)
Minority ethnic groups	374 (28.1%)	1.25 (1.09–1.42)	455 (48.3%)	1.27 (1.10–1.46)	723 (59.7%)	1.30 (1.14–1.47)
Not known	417 (10.2%)	0.47 (0.42–0.53)	581 (19.5%)	0.40 (0.36–0.45)	1287 (31.5%)	0.43 (0.39–0.47)
NHS board of residence						
Greater Glasgow and Clyde	1532 (23.3%)	1.00	1647 (37.7%)	1.00	2361 (44.1%)	1.00
Lothian	1129 (20.1%)	0.84 (0.76–0.92)	1253 (35.0%)	0.89 (0.81–0.98)	2220 (51.1%)	1.52 (1.39–1.66)
Rest of Scotland	1675 (19.8%)	0.82 (0.75–0.89)	1828 (34.5%)	0.93 (0.85–1.02)	3069 (43.0%)	1.11 (1.03–1.20)
Deprivation quintile of residence						
1–2 (most deprived)	1895 (22.4%)	1.01 (0.94–1.08)	2051 (35.9%)	0.94 (0.87–1.01)	3164 (44.6%)	0.92 (0.86–0.98)
3–5	2336 (21.1%)	1.00	2585 (36.2%)	1.00	4285 (46.8%)	1.00
Not known	105 (9.3%)	0.52 (0.42–0.64)	92 (22.5%)	0.62 (0.48–0.79)	201 (34.0%)	0.67 (0.56–0.81)
Ever injected drugs						
No	3950 (21.4%)	1.00	4316 (36.3%)	1.00	6935 (46.1%)	1.00
Yes	142 (34.8%)	1.60 (1.29–2.00)	115 (39.8%)	0.97 (0.75–1.24)	143 (46.7%)	0.95 (0.75–1.21)
Not known	244 (13.5%)	0.75 (0.65–0.87)	297 (27.3%)	0.79 (0.68–0.92)	572 (38.9%)	0.80 (0.71–0.90)
Recent STI/PEP history^c^						
Diagnosed with a rectal STI and/or prescribed HIV PEP	1140 (39.9%)	2.94 (2.69–3.21)	1070 (51.1%)	2.10 (1.90–2.32)	1848 (60.1%)	1.82 (1.67–1.98)
Diagnosed with an STI (non‐rectal)	360 (24.8%)	1.47 (1.29–1.67)	366 (45.6%)	1.62 (1.39–1.88)	625 (45.5%)	1.10 (0.98–1.24)
Not diagnosed with an STI or prescribed PEP	2836 (17.3%)	1.00	3292 (31.8%)	1.00	5177 (41.8%)	1.00

^a^ Relates to the period prior to a patient's first attendance within each analysis period going back to the commencement of NaSH in 2008.

^b^ White British includes White with unspecified nationality; White Other includes minority White ethnic groups and people of non‐British White ethnicity.

^c^ Within a year prior to the date of first attendance in a period or up to 3 months beyond the date of first attendance. STI only includes gonorrhoea and chlamydia. Patients have been assigned to only one category based on their highest risk factor.

^d^ Denominators used to derive the percentages prescribed PrEP are shown in Table 1.

^e^ Adjusted for all covariates listed in the table.

Compared to those with no recent STI/PEP history, those prescribed PEP and/or with a rectal STI diagnosis were more likely to have been prescribed PrEP in PrEP period 1 (aOR 2.94, 95% CI 2.69–3.21), as were those with a non‐rectal STI diagnosis (aOR 1.47, 95% CI 1.29–1.67). For the same comparisons in PrEP period 2, results were similar (aOR 2.10, 95% CI 1.90–2.32 and aOR 1.62, 95% CI 1.39–1.88, respectively); in PrEP period 3, those prescribed PEP and/or with a rectal STI diagnosis had under two‐fold odds of having been prescribed PrEP (aOR 1.82, 95% CI 1.67–1.98) while there was less evidence of an increased odds for those with a non‐rectal STI diagnosis (aOR 1.10, 95% CI 0.98–1.24).

Some factors had differing associations with being prescribed PrEP over time. In PrEP period 1, GBMSM aged 50+ were more likely to be prescribed PrEP than those aged <24 (aOR 1.19, 95% CI 1.05–1.34) but there was no difference between these groups in PrEP periods 2 and 3 (aOR 1.04, 95% CI 0.91–1.19 and aOR 0.93, 95% CI 0.83–1.05, respectively). While having a history of injecting drug use was associated with PrEP coverage in PrEP period 1 (aOR 1.60, 95% CI 1.29–2.00), this was no longer significant in PrEP period 2 (aOR 0.97, 95% CI 0.75–1.24) and PrEP period 3 (aOR 0.95, 95% CI 0.75–1.21).

### HIV incidence and effect of PrEP

**FIGURE 1:**
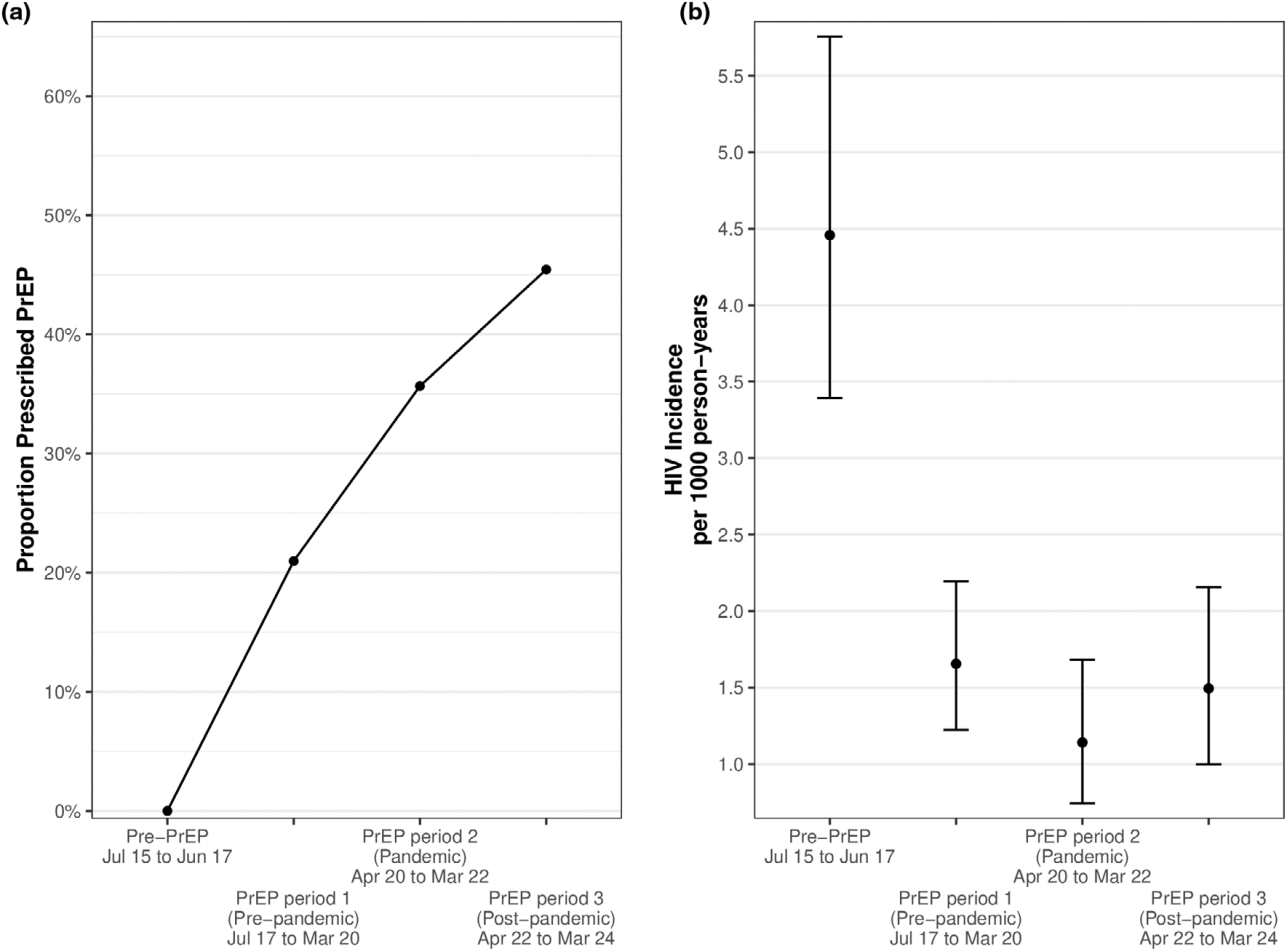
Proportion of GBMSM patients attending sexual health clinics in Scotland that were prescribed HIV PrEP by calendar period of exposure (a) and HIV incidence per 1000 person‐years with 95% confidence intervals by calendar period of exposure (b).

Over the study period, there were 149 incident HIV infections among 21 607 patients followed up for 77 078 PY, representing an incidence rate of 1.93 per 1000 PY (95% CI 1.64–2.26) (Table 3). The incidence rate decreased from the pre‐PrEP period (4.46, 95% CI 3.39–5.76) to PrEP period 1 (1.66, 95% CI 1.22–2.19) but remained relatively stable thereafter (PrEP period 2 1.14, 95% CI 0.74–1.68; PrEP period 3 1.49, 95% CI 1.00–2.16). Compared to the pre‐PrEP period, the risk of HIV acquisition was lower in all subsequent periods (PrEP period 1 aIRR 0.37, 95% CI 0.25–0.54; PrEP period 2 aIRR 0.25, 95% CI 0.15–0.41; PrEP period 3 aIRR 0.30, 95% CI 0.19–0.48). GBMSM diagnosed with, compared to without, an STI or prescribed PEP had a greater risk of acquiring HIV (aIRR 2.14, 95% CI 1.54–2.97); this group also experienced the largest decrease in incidence rate over time (pre‐PrEP 8.97 per 1000 PY, 95% CI 6.14–12.71; PrEP period 1 2.36, 95% CI 1.40–3.74; PrEP period 2 1.88, 95% CI 0.93–3.42; PrEP period 3 2.11, 95% CI 1.12–3.66) (Figure 1). People of a minority ethnic group and people with a history of injecting drugs had an increased risk of acquiring HIV (aIRR 2.06, 95% CI 1.25–3.39 and aIRR 3.52, 95% CI 1.94–6.39, respectively).

**TABLE 3:** HIV incidence per 1000 person‐years and relative risks for incident HIV acquisition by calendar period of PrEP exposure.

	Patients	Person years	Incident HIV acquisitions	Incidence (95% CI)	Unadjusted incidence RR (95% CI)	Adjusted incidence RR (95% CI)
Total	21 607	77 078	149	1.93 (1.64–2.26)		
PrEP exposure period (time dependent)^a^						
Pre‐PrEP: Jul 2015 to Jun 2017	9879	12 338	55	4.46 (3.39–5.76)	1.00	1.00
PrEP period 1: Jul 2017 to Mar 2020	15 000	27 186	45	1.66 (1.22–2.19)	0.37 (0.25–0.55)	0.37 (0.25–0.54)
PrEP period 2: Apr 2020 to Mar 2022	12 397	20 158	23	1.14 (0.74–1.68)	0.26 (0.16–0.42)	0.25 (0.15–0.41)
PrEP period 3: Apr 2022 to Mar 2024	12 579	17 396	26	1.49 (1.00–2.16)	0.34 (0.21–0.53)	0.30 (0.19–0.48)
Age						
<24	7661	26 681	50	1.87 (1.41–2.45)	1.00	1.00
25–39	9387	33 472	68	2.03 (1.59–2.56)	1.08 (0.75–1.56)	1.06 (0.73–1.53)
40+	4559	16 926	31	1.83 (1.27–2.57)	0.98 (0.62–1.53)	1.05 (0.67–1.65)
Ethnicity^c^						
White British	14 447	58 465	99	1.69 (1.38–2.05)	1.00	1.00
White Other	1579	5101	7	1.37 (0.61–2.69)	0.81 (0.38–1.74)	0.76 (0.35–1.66)
Minority ethnic groups	1635	5527	19	3.44 (2.14–5.26)	2.03 (1.24–3.32)	2.06 (1.25–3.39)
Not known	3946	7986	24	3.01 (1.98–4.40)	1.77 (1.14–2.77)	2.14 (1.36–3.37)
NHS board of residence						
Greater Glasgow and Clyde	7396	27 516	51	1.85 (1.40–2.42)	1.00	1.00
Lothian	5568	19 730	44	2.23 (1.64–2.96)	1.20 (0.80–1.80)	1.30 (0.86–1.98)
Rest of Scotland	8643	29 832	54	1.81 (1.37–2.34)	0.98 (0.67–1.43)	1.11 (0.75–1.64)
Deprivation quintile of residence^b^						
1–2 (most deprived)	9051	33 783	71	2.10 (1.65–2.63)	1.00	1.00
3–5	12 556	43 295	78	1.80 (1.43–2.24)	0.86 (0.62–1.18)	0.86 (0.62–1.20)
Ever injected drugs^b^						
No	21 218	75 408	137	1.82 (1.53–2.14)	1.00	1.00
Yes	389	1670	12	7.19 (3.92–12.17)	3.95 (2.19–7.13)	3.52 (1.94–6.39)
Recent STI/PEP history^d^						
Diagnosed with an STI or prescribed PEP^e^	8536	20 011	65	3.25 (2.53–4.11)	2.21 (1.60–3.05)	2.14 (1.54–2.97)
Not diagnosed with an STI or prescribed PEP	18 787	57 067	84	1.47 (1.18–1.81)	1.00	1.00

^a^ Individuals can contribute to multiple exposure periods.

^b^ A small number of patients with unknown deprivation were assumed to be quintiles 3–5, and those with unknown injection history were assumed to have never injected.

^c^ White British includes White individuals with unspecified nationality; White Other includes minority White ethnic groups and people of non‐British White ethnicity.

^d^ Individuals can contribute to multiple risk groups. These were assigned to individuals separately for each analysis period to which they contributed person‐time.

^e^ Includes patients who had an STI (gonorrhoea or chlamydia) or were prescribed HIV PEP within 12 months prior to their first attendance in each analysis period or up to 3 months after their first attendance in each period.

**FIGURE 2:**
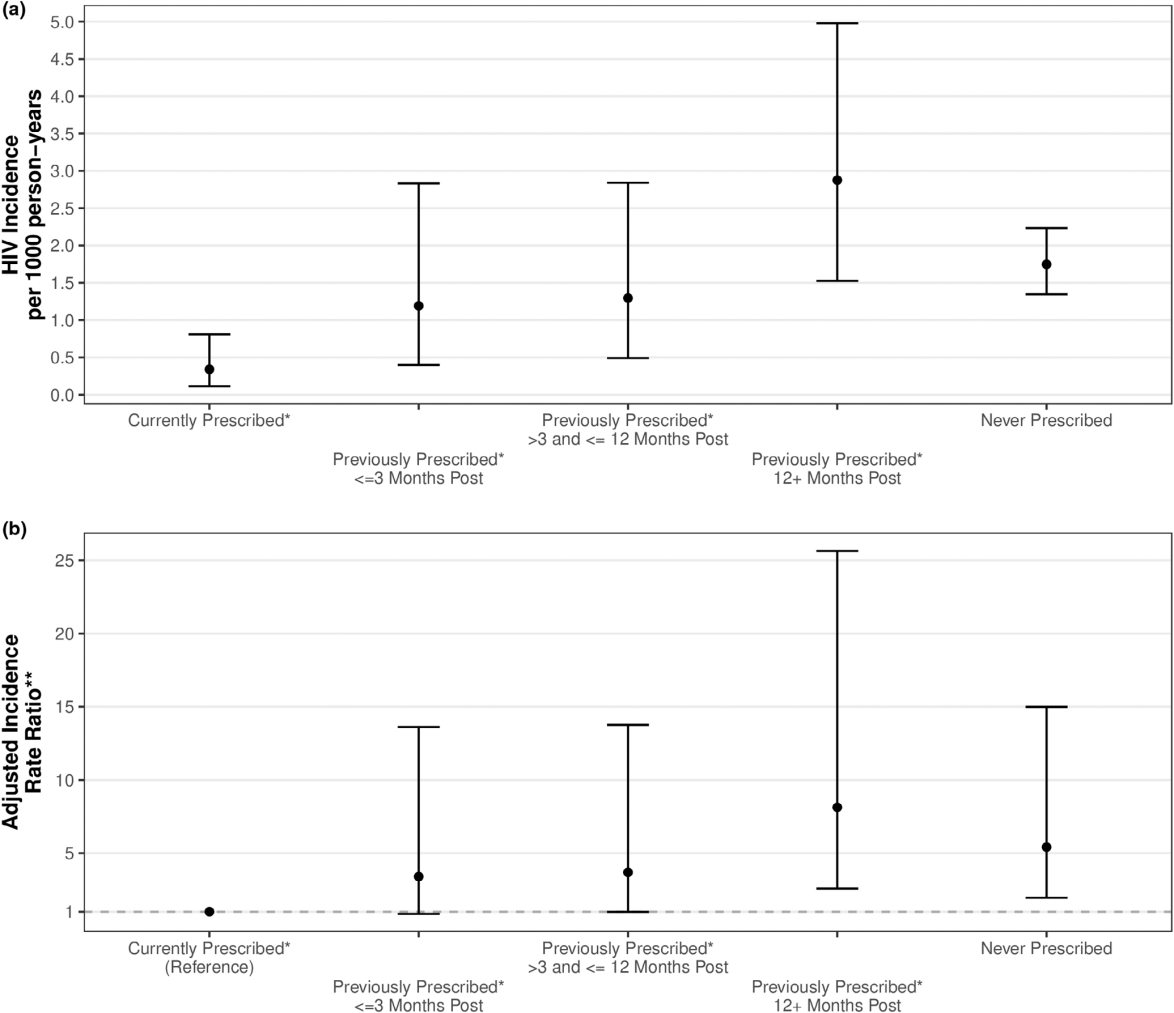
HIV incidence per 1000 person‐years (a) and adjusted incidence rate ratios for incident HIV infection (b) since the start of the PrEP programme (July 2017) up to March 2024 by prescribed PrEP status. * ‘Currently Prescribed’ refers to periods where patients were assumed to have sufficient PrEP supply, assuming daily use for prescriptions where event‐based use was not indicated and assuming PrEP was used four times each week where event‐based use was indicated; ‘Previously Prescribed’ refers to periods after their first ever PrEP prescription outwith those covered by ‘Currently Prescribed’. ** Also adjusted for age, ethnicity, NHS board of residence, deprivation quintile, ever injected drugs and recent STI history.

In analyses confined to the PrEP period, there were 84 HIV acquisitions among 19 012 patients followed up for 57 086 PY (incidence rate of 1.47 per 1000 PY, 95% CI 1.18–1.81) (Table 4). Four HIV acquisitions occurred during periods when patients were considered to be currently prescribed PrEP (incidence rate 0.34 per 1000 PY, 95% CI 0.11–0.81). Currently prescribed was associated with a reduced risk compared to never being prescribed (aIRR 0.19, 95% CI 0.07–0.51). The incidence rate for those never prescribed (1.75, 95% CI 1.35–2.23) was similar to those previously prescribed (1.81, 95% CI 1.14–2.74; aIRR 0.95, 95% CI 0.57–1.59). However, incidence rates increased with longer duration since last PrEP prescription (<3 months post: 1.19 per 1000 PY, 95% CI 0.40–2.83; 3–12 months post: 1.30 per 1000 PY, 95% CI 0.49–2.84; 12+ months post: 2.88 per 1000 PY, 95% CI 1.53–4.98) (Figure 2). Compared to those currently prescribed, there was an increased risk of HIV acquisition in the period of more than one year since the last prescription (aIRR 8.13, 95% CI 2.58–25.64). Sensitivity analysis which applied different assumptions to daily and event‐based prescriptions resulted in minor changes to estimates (Table S4).

**TABLE 4:** HIV incidence per 1000 person‐years and relative risks for incident HIV acquisition since the start of the PrEP programme (July 2017) up to March 2024 by prescribed PrEP status.

	Patients	Person years	Incident HIV acquisitions	Incidence (95% CI)	Unadjusted incidence RR (95% CI)	Adjusted incidence RR (95% CI)^c^
Total	19 012	57 086	84	1.47 (1.18–1.81)		
PrEP status (time dependent)^a^						
Never prescribed	17 962	34 328	60	1.75 (1.35–2.23)	1.00	1.00
Previously prescribed^b^	7899	11 038	20	1.81 (1.14–2.74)	1.04 (0.62–1.72)	0.95 (0.57–1.59)
Currently prescribed^†^	9410	11 720	4	0.34 (0.11–0.81)	0.20 (0.07–0.54)	0.19 (0.07–0.51)

^a^ Individuals can contribute to multiple PrEP status categories.

^b^ 'Currently Prescribed' refers to periods where patients were assumed to have sufficient PrEP supply, assuming daily use for prescriptions where event‐based use was not indicated and assuming PrEP was used four times each week where event‐based use was indicated; 'Previously Prescribed' refers to periods after their first ever PrEP prescription outwith those covered by 'Currently Prescribed'.

^c^ Also adjusted for age, ethnicity, NHS board of residence, deprivation quintile, ever injected drugs and recent STI history.

## DISCUSSION

### Main findings

This study extends previous analysis evaluating the first two years of the Scottish PrEP programme [6] and examines HIV incidence over almost seven years for a cohort of nearly 22 000 GBMSM. This is estimated to relate to nearly one‐third of all GBMSM in Scotland [22] and provides considerably more data to evaluate the national PrEP programme. We report evidence of a sustained effect of the programme in maintaining a low HIV incidence of under two acquisitions per 1000 PY among GBMSM attending sexual health clinics, including throughout the COVID‐19 pandemic. These incidence estimates represent one of the lowest rates reported among a national cohort of GBMSM in a country with historically higher incidence (in excess of four HIV acquisitions per 1000) prior to the introduction of PrEP. However, despite (i) an increase in PrEP coverage from 21% in the initial PrEP period to 45% in the most recent period and (ii) strong evidence of a protective effect of PrEP, further reductions in HIV incidence were not observed. Furthermore, we found evidence of an elevated risk of incident HIV acquisition for those with one year lapsed since their last prescription, likely relating to a period when individuals were no longer using PrEP.

There are several possible explanations for the lack of a further reduction in HIV incidence despite a doubling of PrEP coverage. These may include changes in testing behaviour, service engagement or risk composition over time. It is likely that many individuals at ‘greater’ risk of acquiring HIV, such as those with a recent STI diagnosis and HIV PEP, were already engaged with sexual health services at the start of the national programme and, therefore, became early PrEP adopters, leading to a pronounced reduction in incidence during that initial period. However, as the programme progressed, the combination of (i) fewer patients at ‘greater’ risk of acquiring HIV newly attending services each year and (ii) PrEP discontinuation among those at ‘greater’ risk makes it more challenging to achieve further reductions in incidence for the population attending services. In addition, changes in prescribing practice and broadening eligibility criteria to a ‘PrEP for all who could benefit’ ethos [23] were introduced in late 2022 [9]. This meant that men initially ineligible for PrEP due to lower reported risk became eligible in later years. Thus, while we observed an overall doubling in PrEP coverage among GBMSM attending clinics over the analysis periods, the scale‐up was greater among those with a lower risk of HIV acquisition: for example, we observed a 2.4‐fold rise in coverage for those without (compared to a 1.5‐fold rise for those with) a recent STI diagnosis or PEP prescription. While PrEP discontinuation is a concern for those at sustained greater risk, it is also a concern more broadly as many individuals can occasionally experience short periods in which they are at higher risk [24].

The low incidence found in this Scottish study is consistent with studies investigating the effectiveness of PrEP in Australia [10, 12]. The latter were confined to those dispensed PrEP, while our Scottish cohort involved GBMSM not prescribed PrEP attending sexual health clinics. The relatively low HIV incidence (1.75 per 1000 PY) observed among those never prescribed PrEP suggests that Scotland's PrEP programme, in combination with changes to treatment guidelines recommending rapid commencement of antiretroviral therapy [25], has acted to reduce community transmission and sustain low HIV incidence among GBMSM more broadly. Other studies from the United Kingdom, Australia and the United States have similarly found combinations of HIV prevention measures, working alongside PrEP, have facilitated reductions in HIV transmission [26, 27, 28, 29].

We estimated that almost 4000 people had discontinued PrEP for at least four months (>35% of those ever prescribed PrEP), with almost 2000 having discontinued PrEP for 12 months or longer. Furthermore, we observed a number of new HIV diagnoses among PrEP‐experienced GBMSM who had stopped receiving PrEP prescriptions, with an elevated risk of HIV in the period beyond one year of the assumed prescription end date (aIRR 8.13, 95% CI 2.58–25.64) compared to the period on PrEP. Our findings are consistent with growing concerns for those who have discontinued PrEP and suggest that further prevention measures—which may include continued engagement and maintenance on PrEP—will be needed to reduce HIV incidence further. A meta‐analysis found that 41% of participants discontinued PrEP within six months but reduced to 25% for studies that had adherence interventions [14].

### Limitations

The retrospective cohort approach required at least two HIV tests to be included in the incidence analysis. Approximately two‐thirds of those with HIV were diagnosed on their first test (Table S5) and thus were not included in incidence analysis. As many HIV diagnoses occurred at first test, HIV incidence estimates may not fully represent GBMSM less engaged with testing services. HIV incidence may differ among GBMSM who did not return for testing; therefore, our estimates may not represent the wider cohort. However, our findings are broadly in line with national surveillance data showing a decline in the overall number of new diagnoses (involving recently‐acquired infection) among GBMSM from 132 (44) in 2015 to 47 (11) in 2024; declines were less pronounced between 2021 and 2024: with a range of 36–49 new diagnoses and a range 6–11 recently‐acquired infections [30, 31]. In addition, administrative data relating to incident syphilis infection, sexual behaviour or condom use were not available, which would help to better stratify patients according to risk. Due to the small numbers involved, analyses did not consider individual ethnic groups separately due to issues of disclosure and model fitting.

We do not have data on how many GBMSM in Scotland have a ‘PrEP‐need’ (including those who have not attended sexual health clinics) and therefore, are unable to assess uptake of PrEP according to overall need. However, a doubling in PrEP coverage to 45% among those who attended clinics suggests that PrEP use is relatively high, particularly when compared with a global systematic review which estimated just over 20% of PrEP‐eligible GBMSM to have current PrEP use [32]. Accurate assessment of PrEP adherence is known to be challenging. Although imperfect, our measure of PrEP adherence using available information on PrEP prescriptions to infer how long patients' PrEP supply should last has some benefits over the use of medication possession ratios (MPRs) [5, 10, 12]. The former approach allows for the classification of person‐years of risk according to periods of PrEP use compared with essentially dividing the cohort by their MPR. Furthermore, sensitivity analyses showed that the results were consistent across varying assumptions of PrEP prescription use.

### Implication

The national PrEP programme has greatly reduced the risk of HIV acquisition among GBMSM in Scotland. However, doubling uptake and broadening coverage of PrEP did not further reduce HIV incidence in this population. This result should be taken in the context that sexual risk activity may have increased within this population since the introduction of PrEP. Notably, concerns around unmet need for condoms were reported by GBMSM during the COVID‐19 pandemic [33] and there has been a significant rise in gonorrhoea and syphilis diagnoses in Scotland since 2022 [34]. Thus, there is a possibility that HIV incidence could have increased without the scale‐up of the PrEP programme in recent years, but we cannot estimate this based on our statistical analysis of observational data alone. Instead, dynamic mathematical modelling work [35] is warranted to help understand how HIV incidence could be further reduced while considering the complexities of changes in sexual risk behaviour and PrEP coverage among different groups of individuals. However, our analysis indicates that HIV incidence could have been further reduced if PrEP persistence was improved.

The elevated risk of HIV acquisition among those previously prescribed PrEP could be attributable to several factors requiring different mitigating approaches. More effort may be needed to provide the tools and motivation for people to assess their own HIV acquisition risk and ensure that they can rapidly access PrEP if needed [36]. People on PrEP may benefit from greater support from services and community interventions to encourage persistence [37, 38, 39, 40], in addition to broadening access through a wider range of settings [41] or adapting service models (e.g. telehealth or online clinical care pathways) [42, 43].

Although we found reductions in HIV incidence associated with HIV PrEP among GBMSM as a whole, these reductions may not have occurred across all subgroups. In other studies, PrEP effectiveness was found to be lower among people aged under 30 and socioeconomically disadvantaged people [7, 44]. While the risk of HIV acquisition did not vary significantly by socioeconomic status in our study, GBMSM resident in the most deprived communities were marginally less likely to have received PrEP in the most recent period (April 2022–March 2024) compared to those living in other areas. We found an increased risk of acquiring HIV for GBMSM from minority ethnic groups compared to those of White British ethnicity. However, this may reflect differences in service engagement and testing. Ethnic minority GBMSM may be less likely to attend clinics as they tend to have greater concerns regarding confidentiality [45]; and so, our findings (confined to those attending clinics) may not reflect the wider population, particularly as those seeking specialist sexual health care may have done so due to factors (such as symptomatic STI) which are also associated with an increased risk of HIV acquisition. Thus, further monitoring and evaluation are warranted in these subgroups.

The proportion of GBMSM diagnosed with HIV following their first test at sexual health clinics has not declined over time, suggesting that these individuals are less engaged with healthcare services offering HIV testing and issues relating to equity of access to PrEP or stigma in certain groups [46]. Improving HIV testing rates in health services other than specialist sexual health services is crucial for improving the diagnosis of HIV, linking to HIV combination prevention measures and reducing HIV transmission.

HIV prevention in the future is likely to take advantage of long‐acting injectable forms of PrEP [47, 48]. These medications could help address unmet needs, with increasing evidence of high acceptability and improved adherence in those with practical and psychosocial barriers to uptake [49, 50]. However, current costs far exceed those of oral PrEP, which will limit implementation. Although long‐acting agents reduce the need for daily adherence, there will still need to be efforts to encourage PrEP persistence and follow up with those who do not return for further doses.

## CONCLUSION

Scotland's PrEP programme is having a sustained effect in reducing HIV transmission in GBMSM, with benefit to those taking PrEP and also those not on PrEP. However, further efforts are needed to maximize PrEP's impact, notably the development of interventions to encourage sustained engagement in PrEP care and re‐engage people previously prescribed PrEP. Additional efforts to increase access to and uptake of HIV testing are needed to ensure we identify and support a greater range of people in need of HIV prevention and PrEP care pathways. The challenges of driving HIV incidence below 1 per 1000 person‐years should not be underestimated.

## AUTHOR CONTRIBUTIONS

AY, CE and SH conceived and designed the study. AY led the data analysis and drafting of the manuscript. CE, RM, AR and SH also contributed substantially to the drafting of the manuscript. All authors interpreted the findings, contributed to manuscript revisions and approved the submitted manuscript.

## FUNDING INFORMATION

This research was supported by Public Health Scotland and the Scottish Government Sexual Health and Blood Borne Viruses Strategy Funding Programme 2023–2026. AY received a research grant from the Scottish Government.

## CONFLICT OF INTEREST STATEMENT

The authors declare that they have no known competing financial interests or personal relationships that could have appeared to influence the work reported in this paper.

## Supporting information


**Figure S1.** Proportion of GBMSM patients attending sexual health clinics in Scotland who were prescribed HIV PrEP by calendar period of exposure and various patient characteristics.

**Figure S2.** Proportion of GBMSM patients attending sexual health clinics in Scotland who were prescribed HIV PrEP by calendar period of exposure (a) and HIV incidence per 1000 person‐years with 95% confidence intervals by analysis period (b), grouped by whether or not patients were diagnosed with an STI or prescribed HIV post‐exposure prophylaxis.

**Table S1.** PrEP prescriptions received by GBMSM patients attending sexual health clinics in Scotland between July 2017 and March 2024, by regimen.
**Table S2.** GBMSM attendance at sexual health clinics in Scotland and percentage prescribed HIV PrEP by history of being prescribed HIV post‐exposure prophylaxis (PEP) and history of being diagnosed with a rectal STI*.
**Table S3.** HIV incidence per 1000 person‐years and relative risks for incident HIV acquisition by analysis period, with the reference period set to PrEP period 1 (prior to the COVID‐19 pandemic).
**Table S4.** Sensitivity analyses for estimation of HIV incidence per 1000 person‐years and relative risks for incident HIV acquisition since the start of the PrEP programme (July 2017) up to March 2024 by prescribed PrEP status, with varying assumptions for daily and event‐based prescriptions.
**Table S5.** Incident HIV acquisitions recorded on NaSH among GBMSM according to their history of testing for HIV in sexual health clinics.

## Data Availability

Data may be obtained from a third party and are not publicly available. Access to de‐identified participant data can be sought through approval of the Public Benefit and Privacy Panel for Health and Social Care (www.informationgovernance.scot.nhs.uk/pbpphsc/home/for-applicants/).
