# Supplementary material for: Sustained effect of Scotland's HIV pre‐exposure prophylaxis programme on transmission among gay and bisexual men who have sex with men: Population‐based retrospective cohort study
Source: HIV Med. 2026 May 8;27(8):1298–311. doi: 10.1111/hiv.70255 (PMC13432567; doi:10.1111/hiv.70255)
Supplement: Supplementary file 3 — Table S1. PrEP prescriptions received by GBMSM patients attending sexual health clinics in Scotland between July 2017 and March 2024, by regimen. Table S2. GBMSM attendance at sexual health clinics in Scotland and percentage prescribed HIV PrEP by history of being prescribed HIV post‐exposure prophylaxis (PEP) and history of being diagnosed with a rectal STI*. Table S3. HIV incidence per 1000 person‐years and relative risks for incident HIV acquisition by analysis period, with the reference period set to PrEP period 1 (prior to the COVID‐19 pandemic). Table S4. Sensitivity analyses for estimation of HIV incidence per 1000 person‐years and relative risks for incident HIV acquisition since the start of the PrEP programme (July 2017) up to March 2024 by prescribed PrEP status, with varying assumptions for daily and event‐based prescriptions. Table S5. Incident HIV acquisitions recorded on NaSH among GBMSM according to their history of testing for HIV in sexual health clinics. [file HIV-27-1298-s002.docx]

**Table S1** PrEP prescriptions received by GBMSM patients attending sexual health clinics in Scotland between July 2017 and March 2024, by regimen.

|  | **Number of Prescriptions** | **Percent** |
| --- | --- | --- |
| **Regimen** |  |  |
| Daily | 41,483 | 77.6% |
| Event-Based | 12,002 | 22.4% |
| **Daily PrEP** |  |  |
| 1 Month | 4,327 | 10.4% |
| 2 Months | 2,174 | 5.2% |
| 3 Months | 25,762 | 62.1% |
| 4 Months | 3,812 | 9.2% |
| 5 Months | 353 | 0.9% |
| 6 Months | 3,940 | 9.5% |
| 7 Months | 1,115 | 2.7% |
| **Event-Based PrEP** |  |  |
| 30 Pills | 3,496 | 29.1% |
| 60 Pills | 4,576 | 38.1% |
| 90 Pills | 3,469 | 28.9% |
| 120 Pills | 461 | 3.8% |

**Table S2** GBMSM attendance at sexual health clinics in Scotland and percentage prescribed HIV PrEP by history of being prescribed HIV post-exposure prophylaxis (PEP) and history of being diagnosed with a rectal STI*.

|  | **Pre-PrEP period (July 2015 to June 2017)** | **PrEP period 1 (July 2017 to March 2020)** | **PrEP period 2 (April 2020 to March 2022)** | **PrEP period 3 period (April 2022 to March 2024)** |
| --- | --- | --- | --- | --- |
| **Percentage of Attendees** |  |  |  |  |
| Prescribed HIV PEP and Diagnosed with a rectal STI^*^ | 51 / 14752 (0.3%) | 114 / 20674 (0.6%) | 62 / 13257 (0.5%) | 106 / 16832 (0.6%) |
| Prescribed HIV PEP (no rectal STI)^*^ | 297 / 14752 (2.0%) | 614 / 20674 (3.0%) | 440 / 13257 (3.3%) | 524 / 16832 (3.1%) |
| Diagnosed with a rectal STI (no HIV PEP)^*^ | 1557 / 14752 (10.6%) | 2127 / 20674 (10.3%) | 1591 / 13257 (12.0%) | 2444 / 16832 (14.5%) |
| **Percentage prescribed PrEP** |  |  |  |  |
| Prescribed HIV PEP and Diagnosed with a rectal STI^*^ | 0 / 51 (0%) | 76 / 114 (66.7%) | 39 / 62 (62.9%) | 82 / 106 (77.4%) |
| Prescribed HIV PEP (no rectal STI)^*^ | 0 / 297 (0%) | 242 / 614 (39.4%) | 190 / 440 (43.2%) | 262 / 524 (50.0%) |
| Diagnosed with a rectal STI (no HIV PEP)^*^ | 0 / 1557 (0%) | 822 / 2127 (38.6%) | 841 / 1591 (52.9%) | 1504 / 2444 (61.5%) |
| ^*^Within a year prior to the date of first attendance in a period or up to 3 months beyond date of first attendance. STI only includes gonorrhoea and chlamydia. | | | | |

**Table S3** HIV incidence per 1000 person-years and relative risks for incident HIV acquisition by analysis period, with reference period set to PrEP period 1 (prior to the COVID-19 pandemic).

|  | **Patients** | **Person Years** | **Incident HIV Acquisitions** | **Incidence (95% CI)** | **Unadjusted Incidence RR (95% CI)** | **Adjusted Incidence RR (95% CI)^†^** |
| --- | --- | --- | --- | --- | --- | --- |
| Total | 21811 | 77671 | 150 | 1.93 (1.64-2.26) |  |  |
| **PrEP Exposure Period (Time Dependent)^*^** |  |  |  |  |  |  |
| Pre-PrEP: Jul 2015 to Jun 2017 | 9916 | 12388 | 55 | 4.44 (3.38-5.73) | 2.70 (1.82-4.00) | 2.72 (1.83-4.04) |
| PrEP period 1: Jul 2017 to Mar 2020 | 15109 | 27359 | 45 | 1.64 (1.22-2.18) | 1.00 | 1.00 |
| PrEP period 2: Apr 2020 to Mar 2022 | 12525 | 20349 | 23 | 1.13 (0.74-1.67) | 0.69 (0.42-1.14) | 0.68 (0.41-1.12) |
| PrEP period 3: Apr 2022 to Mar 2024 | 12741 | 17575 | 27 | 1.54 (1.04-2.20) | 0.93 (0.58-1.51) | 0.85 (0.53-1.37) |
| ^*^Individuals can contribute to multiple exposure periods. | | | | | | |
| ^†^Also adjusted for age, ethnicity, NHS board of residence, deprivation quintile, ever injected drugs and recent STI history. | | | | | | |

**Table S4** Sensitivity analyses for estimation of HIV incidence per 1000 person-years and relative risks for incident HIV acquisition since the start of the PrEP programme (July 2017) up to March 2024 by prescribed PrEP status, with varying assumptions for daily and event-based prescriptions.

|  | **Patients^*^** | **Person Years** | **Incident HIV Acquisitions** | **Incidence (95% CI)** | **Unadjusted Incidence RR (95% CI)** | **Adjusted Incidence RR (95% CI)^†^** |
| --- | --- | --- | --- | --- | --- | --- |
| **Daily: 5 pills/week Event-based: 2 pills/week** |  |  |  |  |  |  |
| Never Prescribed | 17962 | 34328 | 60 | 1.75 (1.35-2.23) | 1.00 | 1.00 |
| Previously Prescribed | 6150 | 8328 | 17 | 2.04 (1.23-3.19) | 1.17 (0.68-2.00) | 1.06 (0.62-1.84) |
| Currently Prescribed | 9453 | 14509 | 7 | 0.48 (0.22-0.95) | 0.28 (0.13-0.60) | 0.26 (0.12-0.58) |
| **Daily: 7 pills/week Event-based: 7 pills/week** |  |  |  |  |  |  |
| Never Prescribed | 17962 | 34328 | 60 | 1.75 (1.35-2.23) | 1.00 | 1.00 |
| Previously Prescribed | 8189 | 11919 | 20 | 1.68 (1.06-2.54) | 0.96 (0.58-1.59) | 0.88 (0.53-1.47) |
| Currently Prescribed | 9411 | 10831 | 4 | 0.37 (0.12-0.88) | 0.21 (0.08-0.58) | 0.20 (0.07-0.56) |
| **Daily: 4 pills/week Event-based: 4 pills/week** |  |  |  |  |  |  |
| Never Prescribed | 17962 | 34328 | 60 | 1.75 (1.35-2.23) | 1.00 | 1.00 |
| Previously Prescribed | 6313 | 9055 | 18 | 1.99 (1.22-3.07) | 1.14 (0.67-1.93) | 1.04 (0.61-1.78) |
| Currently Prescribed | 9453 | 13777 | 6 | 0.44 (0.18-0.90) | 0.25 (0.11-0.58) | 0.24 (0.10-0.55) |
| ^*^Individuals can contribute to multiple PrEP status categories. | | | | | | |
| ^†^Also adjusted for age, ethnicity, NHS board of residence, deprivation quintile, ever injected drugs and recent STI history. | | | | | | |

**Table S5** Incident HIV acquisitions recorded on NaSH among GBMSM according to their history of testing for HIV in sexual health clinics.

|  | **Included in Cohort for HIV Incidence Analysis** | **Pre-PrEP period (July 2015 to June 2017)** | **PrEP period 1 (July 2017 to March 2020)** | **PrEP period 2 (April 2020 to March 2022)** | **PrEP period 3 (April 2022 to March 2024)** |
| --- | --- | --- | --- | --- | --- |
| Tested at least twice for HIV within the study period, or tested within 2 years before first attendance in the study period and then tested positive at first attendance | Yes | 55 (30%) | 45 (24%) | 23 (24%) | 26 (26%) |
| Tested for HIV more than 2 years before first attendance in the study period and then tested positive at first attendance in the study period | No | 15 (8%) | 19 (10%) | 12 (13%) | 10 (10%) |
| Never previously tested for HIV and then tested positive at first attendance in the study period | No | 113 (62%) | 122 (66%) | 59 (63%) | 64 (64%) |
| Total |  | 183 | 186 | 94 | 100 |
